# The association between the metabolic score for insulin resistance and mortality in patients with cardiovascular disease: a national cohort study

**DOI:** 10.3389/fendo.2024.1479980

**Published:** 2024-12-18

**Authors:** Xiaozhou Su, Huiqing Rao, Chunli Zhao, Xianwei Zhang, Donghua Li

**Affiliations:** ^1^ Department of Cardiology, Minzu Affiliated Hospital of Guangxi Medical University, Nanning, China; ^2^ Department of Internal Medicine, Guangxi Medical University Cancer Hospital, Nanning, China

**Keywords:** metabolic score for insulin resistance, mortality, insulin resistance, cardiovascular disease, NHANES

## Abstract

**Background:**

The metabolic score for insulin resistance (METS-IR) is a novel index for evaluating insulin resistance and identifying high-risk cardiovascular disease (CVD) patients. This study aims to assess the prognostic value of METS-IR in predicting mortality risk in CVD patients.

**Methods:**

We analyzed data from 2,515 CVD patients in the National Health and Nutrition Examination Survey (NHANES). Associations between METS-IR and all-cause mortality and cardiovascular mortality were evaluated using multivariable Cox proportional hazards models and restricted cubic splines (RCS). Threshold effects and sensitivity analyses were conducted to ensure robustness.

**Results:**

Over a median follow-up of 91.4 months, 1,090 patients died, including 447 from cardiovascular causes. A U-shaped relationship was identified between lnMETS-IR and all-cause and cardiovascular mortality, with thresholds at 3.70 and 3.67. Below thresholds, an increase of lnMETS-IR was associated with a 75% reduction in the risk of all-cause mortality (HR: 0.25, 95% CI: 0.14–0.46) and a 79% reduction in the risk of cardiovascular mortality (HR: 0.21, 95% CI: 0.07–0.56). While above thresholds, an increase of lnMETS-IR was associated with a 180% increase in the risk of all-cause mortality (HR: 2.80, 95% CI: 1.61–4.88) and a 233% increase in the risk of cardiovascular mortality (HR: 3.33, 95% CI: 1.43–7.75).

**Conclusions:**

This study identified a U-shaped association between lnMETS-IR and mortality among CVD patients, underscoring the potential of METS-IR as a valuable prognostic marker for mortality risk in patients with CVD.

## 1 Introduction

Cardiovascular disease (CVD) remains a significant public health concern and is the primary cause of morbidity and mortality among the major non-communicable diseases, despite significant progress in prevention and management of CVD being made over the past half-century. Data from the AHA Heart Disease and Stroke Statistical Update indicate that approximately 28.6 million American adults were affected by some form of CVD between the late 2010s and 2020 (1). Concurrently, CVD accounted for approximately 18.6 million deaths globally in 2019, representing nearly one-third of global mortality, with approximately 870,000 of those deaths occurring in the U.S (2). This number is predicted to increase to more than 23.6 million people who will die annually by 2030 (3). Consequently, there is an urgent need to identify CVD patients at high mortality risk and to develop clinical interventions aimed at preventing adverse outcomes.

Insulin resistance (IR) serves as a component of cardiovascular metabolic abnormalities, which are commonly referred to as “IR syndrome” or “metabolic syndrome (MetS).” IR accelerates the development of atherosclerosis and hypertension (4) and is closely associated with diabetes (5), stroke (6), and coronary heart disease (7). A growing body of evidence suggests that IR is closely linked to increased mortality risk, including both all-cause and cardiovascular mortality. However, the relationship between IR and mortality risk is not uniform across all population groups, and significant differences have been observed based on age, gender, and ethnicity. The association between IR and mortality risk appears to be particularly pronounced in elderly populations (8), postmenopausal women (9), and African Americans and Hispanics (10). These findings highlight the need to consider population-specific differences when evaluating the risk posed by insulin resistance. Understanding how IR contributes to mortality risk in various demographic groups may help refine risk assessment strategies and develop targeted interventions for reducing the burden of CVD and premature mortality.

The hyperinsulinemic-euglycemic clamp test (HEC) and the homeostasis model assessment of insulin resistance (HOMA-IR) are common techniques for determining IR (11). However, due to the complexity and cost of the process, as well as the fact that insulin levels are not routinely measured in clinical practice, both approaches are ineffective for large-scale epidemiological studies. Hence, some non-insulin-based IR metrics, such as the triglyceride to high-density lipoprotein cholesterol (TG/HDL-c) ratio and the triglyceride-glucose index (TyG index), have been developed. However, existing indices do not account for the effects of nutritional intake and metabolic status, such as body mass index (BMI), thereby limiting the development of effective clinical disease prediction models. METS-IR, a novel index for assessing insulin resistance (IR), strongly correlates with HEC (12). It combines fasting blood glucose (FBG), triglycerides (TG), high-density lipoprotein (HDL-c) and BMI, offering a cost-effective and accessible alternative for large-scale screening and ongoing patient monitoring. Previous studies have demonstrated that METS-IR is closely associated with hypertension (13) and hyperuricemia (14). Additionally, METS-IR is more effective at identifying people developing MetS and T2DM at an early stage than other insulin-dependent indices (15). Emerging evidence suggests that METS-IR is also linked to important cardiovascular indicators, such as coronary artery calcification and subclinical myocardial injury (16), which are early markers of cardiovascular damage. This association highlights the broader impact of METS-IR on cardiovascular health and its potential to predict adverse cardiovascular outcomes. In rural China, baseline METS-IR levels and their long-term trends have been strongly associated with increased risks of CHD and stroke (17). Furthermore, METS-IR has demonstrated superior accuracy in predicting diabetes at various future time intervals in the Japanese population, compared to the TyG index, TyG-BMI, and TG/HDL-c ratio (18). METS-IR has also shown a more significant association with all-cause and cardiovascular mortality in the U.S. population compared to the TyG index, TG/HDL-c, and HOMA-IR (19). As an innovative tool for assessing IR, METS-IR utilizes multiple clinical parameters without the need for fasting insulin, making it more accessible for primary care, community settings, and large-scale studies. By incorporating BMI and HDL-c, METS-IR provides a comprehensive view of metabolic health and facilitates early cardiovascular risk detection, suggesting that it could serve as a superior risk stratification tool in clinical practice. However, its association with mortality in individuals with CVD remains unexplored. We hypothesize that METS-IR will be a significant predictor of mortality in individuals with CVD. This hypothesis is based on the ability of METS-IR to integrate critical metabolic parameters, potentially offering useful prognostic value in CVD individuals. Hence, we conducted this study to investigate the prognostic value of METS-IR for mortality risk in CVD patients.

## 2 Materials and methods

### 2.1 Study design and participants

The National Health and Nutrition Examination Survey (NHANES) is a population-based, cross-sectional survey designed to collect information on the health and nutritional status of the U.S. household population. Using a multistage probability sampling methodology, the data were collected through laboratory tests, in-home structured interviews, and mobile center physical examinations. This research was conducted in accordance with the guidelines outlined in the Declaration of Helsinki. Ethics approval for the study was obtained from the Ethics Committee of the National Center for Health Statistics. Written informed consent was provided by all participants prior to their involvement in this study.

We collected data from participants interviewed between 1999 and 2018. Subsequently, a longitudinal follow-up cohort was created within the NHANES dataset as a result of using the National Death Index (NDI) from the National Center for Health Statistics (NCHS) to ascertain the participants’ survival outcomes. Individuals was diagnosed with CVD if they indicated in a validated questionnaire that they had ever been informed by a physician or other health care provider that they had congestive heart failure, coronary heart disease, angina pectoris, or stroke. More detailed information about self-report questionnaires can be viewed on this website at https://www.cdc.gov/nchs/data.

**Figure 1:**
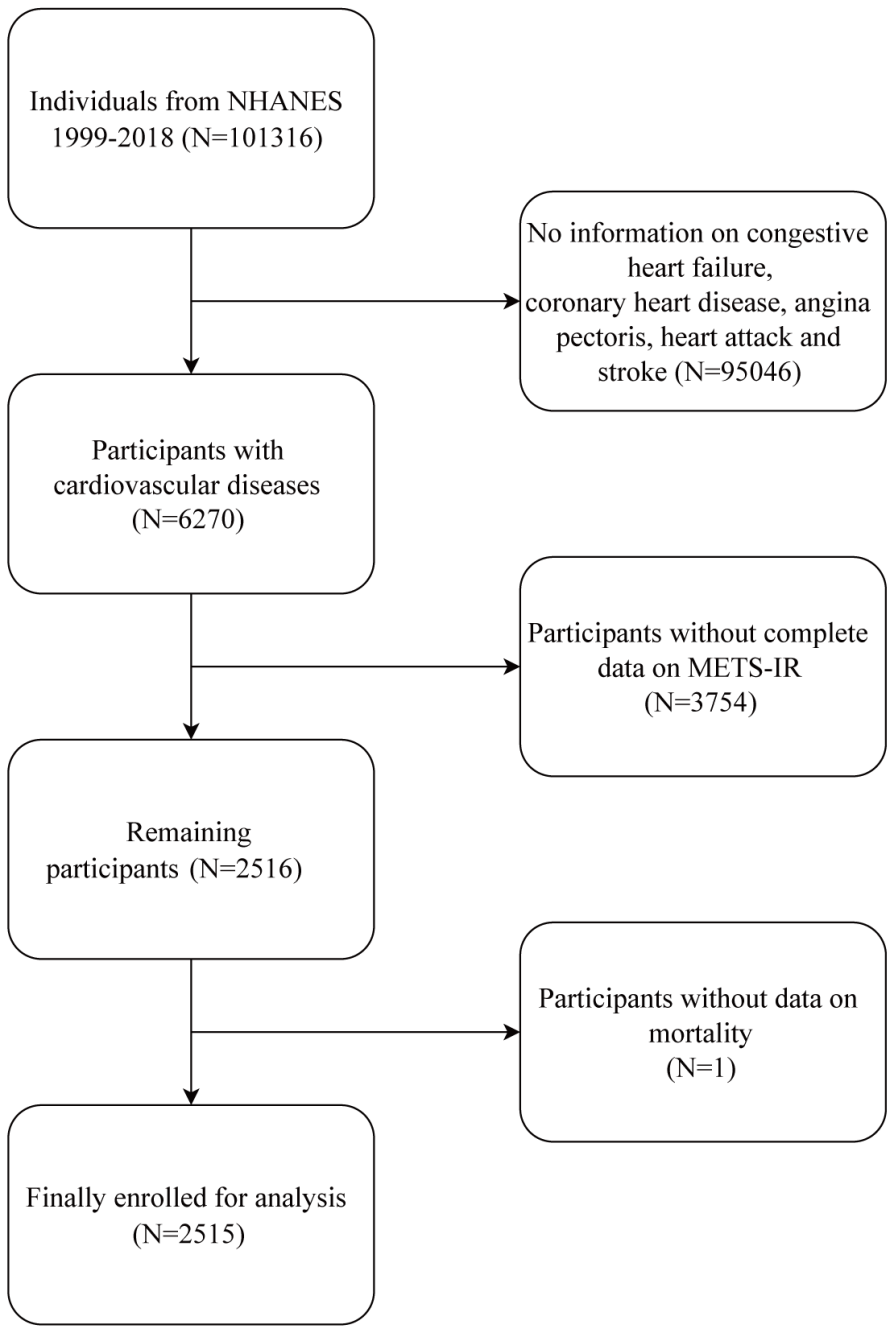
Flow chart of participant selection.

After removing respondents who did not provide baseline information on FBG, TG, HDL-c, or BMI or who did not provide follow-up information, a cohort of 2515 patients diagnosed with CVD was chosen for the study. **Figure 1** depicts the complete data selection procedure. After confirming that each participant gave their informed consent, the NCHS Ethics Committee approved the survey.

### 2.2 Measurement of METS-IR

The METS-IR was computed using the following formula (12): ln(2 × FBG [mg/dL] + TG [mg/dL]) × BMI (kg/m^2)/ln(HDL-c [mg/dL]). Blood parameters were obtained from venous blood samples collected from participants after an overnight fast of at least 8 hours. FBG and TG levels were enzymatically analyzed using a Roche Modular P chemistry analyzer. BMI was calculated using a formula based on height and weight.

### 2.3 Study outcomes

The ICD10 code was used to define the outcome variables, which included all-cause and cardiovascular mortality. Follow-up time was calculated from the date of the NHANES examination to the date of death or December 31, 2019, whichever occurred first. All-cause mortality was determined using the records from NDI, which were connected to the NHANES datasets. Deaths attributed to heart diseases (ICD-10 codes I00-I09, I11, I13, I20-I51) or cerebrovascular diseases (ICD-10 codes I60-I69) were categorized as cardiovascular mortality (20). More detailed information about mortality factors can be viewed on this website at https://www.cdc.gov/nchs/data-linkage/mortality.htm.

### 2.4 Covariates

The participants’ demographic information, medical history, and results from laboratory blood tests were collected. Age, gender, race, education level, marital status, and income-to-poverty ratio (PIR) were among the demographic data. Race/ethnicity was classified into five groups: non-Hispanic White, non-Hispanic Black, Mexican American, other Hispanic, and other races. The three categories of education levels were below high school, high school or equivalent, and college or above. Marital status was categorized as living with a partner or not. Every participant completed a thorough questionnaire about their medical history and lifestyle choices. Based on their smoking status, the participants were divided into three groups: never smoked, former smokers, and current smokers. Individuals who consumed at least 12 drinks within the past 12 months were identified as alcohol users. A questionnaire about participation in vigorous or moderate work or recreational activities was used to measure physical activity (PA). Medical history information included hypertension, diabetes, CVD, and cancer. The criteria for hypertension included a self-reported history of hypertension, oral antihypertensive medication use, or a systolic blood pressure of at least 140 mmHg or a diastolic blood pressure of at least 90 mmHg. The American Diabetes Association (ADA) diagnostic criteria for diabetes state that diabetes can be identified by glycated hemoglobin A1c (HbA1c) ≥ 6.5 percent, fasting glucose ≥ 7.0 mmol/L, use of insulin or oral hypoglycemic medication, or self-reported diagnosis (21). Heart attack, stroke, angina, coronary heart disease, and self-reported heart failure were all included in the CVD history. Laboratory blood test data included fasting glucose, fasting insulin, high-density lipoprotein cholesterol (HDL-c), low-density lipoprotein cholesterol (LDL-c), total cholesterol (TC), TG, HbA1c, serum creatinine, eGFR (estimate glomerular filtration rate), and serum uric acid. Measurements were made to determine BMI, SBP, and DBP. The eGFR calculation in this study was based on the Chronic Kidney Disease Epidemiology Collaboration (CKD-EPI) equation (22).

### 2.5 Statistical analysis

We used the fasting subsample weights and made adjustments to account for multiple cycles. Frequencies and percentages are used to represent categorical variables, and weighted means with standard error are used to represent continuous variables. For the two cycles from 1999 to 2002, the fasting subsample Mobile Examination Center (MEC) weight for 4-year was multiplied by 1/5 to determine the sample weights; for the eight cycles from 2003 to 2018, the fasting subsample 16-year MEC weight was multiplied by 4/5. The METS-IR values underwent logarithmic transformation (ln METS-IR) to account for left skewness. Based on ln METS-IR quartiles (Q1–Q4), four groups of study participants were identified: quartile 1 (<3.624), quartile 2 (3.624-3.791), quartile 3 (3.791-3.978), and quartile 4 (>3.978). A one-way ANOVA was used for continuous variables and a Pearson chi-square test was used for categorical variables in order to compare baseline characteristics between quartile groups. A multivariate Cox regression model was employed to further investigate the impact of METS-IR levels on the risk of all-cause and cardiovascular mortality in CVD patients, with outcomes reported as hazard ratios (HRs) and 95% confidence intervals (CIs). The selection of potential covariates for the multivariable regression models was based on the following criteria: (1) relevant demographic characteristics; (2) variables shown to affect METS-IR and CVD in previous studies (19); (3) variables whose inclusion resulted in a change of more than 10% in the coefficients of the basic model, in accordance with the STROBE statement (23), the basic model changes by more than 10% after the introduction of covariates; and (4) other variables accumulated from clinical experience, including factors that could influence the outcomes but were not captured in the previous categories. To verify that the proportional hazards assumption was satisfied, we conducted the Schoenfeld residual test (**Supplementary Figures S1**, **S2**). This test assesses whether the hazard ratios are proportional over time for each covariate in the model. In Model 1, there was no adjustment; in Model 2, age, gender, and race were adjusted for; in Model 3, variables such as age, gender, race, education level, smoking status, married status, alcohol drinking, BMI, waist circumference, PIR, PA, LDL-c, TC, HbA1c, eGFR, hypertension, and diabetes status were included. To assess the nonlinear relationship between METS-IR and mortality, multivariable restricted cubic splines (RCS) were employed. We chose 3 assumed knots based on the recommendations of Harrell (24), the complexity of the relationship between METS-IR and mortality, Akaike Information Criterion (AIC) (25), and prior similar studies (26). Additionally, the relationship between METS-IR and mortality was examined using a two-piecewise Cox proportional hazards model on either side of the inflection point. Stratified analyses were also performed by age (< 60 or ≥ 60 years), gender (male or female), BMI (< 25, 25-30, or ≥ 30 kg/m^2^), hypertension (yes or no), diabetes (yes or no), physical activity (yes or no), and smoker (current, former, or never). Missing covariate data were imputed via the application of multiple imputation (MI) using chained equation (MICE) methodology. Details of the proportions of missing covariates and the methodology of MI are presented in **Supplementary Table S1**. Two sensitivity analyses were conducted to assess model stability. To minimize the impact of missing data on the main results, participants with missing values were removed from consideration. In addition, participants who were diagnosed with cancer at the start of the study were excluded to reduce potential confounding effects. All statistical analyses were performed using R software (version 4.3.1) and EmpowerStats software. Statistical significance was defined as a two-tailed P-value < 0.05.

## 3 Results

### 3.1 Baseline characteristics

**Table 1** shows the baseline characteristics of participants (n=2515) stratified by lnMETS-IR quartile. The average age of participants was 66.65 years, with 56.10% were male. The mean METS-IR was 46.36 ± 12.82. During a median follow-up of 91.44 months, the rates of all-cause mortality and cardiovascular mortality were 43.34% and 17.77%, respectively. As shown in **Table 1**, compared to patients in the lowest quartile, those with higher lnMETS-IR were typically younger, predominantly male, and had a higher prevalence of hypertension, diabetes, and heart failure. They also demonstrated lower levels of HDL-c, but higher waist circumference, BMI, fasting glucose, fasting insulin, triglycerides, HbA1c, and serum uric acid (all P < 0.05).

**Table 1:** Baseline characteristics of the study individuals according to the lnMETS-IR quartiles.

Variable^a^	Overall	lnMETS-IR Quartiles				P-value
		Q1 (<3.624)	Q2 (3.624-3.791)	Q3 (3.791-3.978)	Q4 (>3.978)	
Participants	2515	629	628	629	629	
Age (year)	64.00 (63.24,64.76)	66.75 (65.15,68.36)	64.57 (62.79,66.36)	64.06 (62.59,65.53)	60.67 (59.34,61.99)	<0.001
Gender (%)						<0.001
Male	54.61 (51.90,57.30)	44.75 (39.48,50.14)	59.30 (53.94,64.46)	59.44 (53.95,64.70)	54.76 (49.00,60.39)	
Female	45.39 (42.70,48.10)	55.25 (49.86,60.52)	40.70 (35.54,46.06)	40.56 (35.30,46.05)	45.24 (39.61,51.00)	
Race (%)						0.061
Mexican American	5.23 (3.91,6.94)	4.78 (2.85,7.92)	3.93 (2.63,5.84)	6.94 (4.33,10.92)	5.16 (3.51,7.55)	
Other hispanic	4.62 (3.46,6.14)	4.72 (2.78,7.88)	6.70 (4.32,10.25)	3.04 (2.07,4.44)	4.11 (2.63,6.37)	
Non-Hispanic White	72.26 (69.05,75.26)	72.39 (67.22,77.03)	72.40 (67.31,76.97)	73.27 (67.72,78.17)	70.96 (66.43,75.11)	
Non-Hispanic Black	11.77 (10.11,13.67)	9.70 (7.50,12.45)	11.01 (8.46,14.20)	12.27 (9.59,15.57)	14.04 (11.26,17.38)	
Other Race	6.13 (4.85,7.72)	8.41 (5.98,11.70)	5.96 (3.90,9.01)	4.49 (2.47,8.01)	5.73 (3.33,9.67)	
Education level (%)						0.332
Less than high school	11.79 (10.01,13.83)	12.80 (9.65,16.78)	12.70 (9.64,16.57)	11.50 (8.67,15.11)	10.19 (7.64,13.47)	
High school or equivalent	43.56 (40.56,46.61)	39.93 (34.93,45.15)	40.61 (35.09,46.37)	47.48 (41.92,53.10)	45.97 (40.16,51.90)	
College or above	44.65 (41.26,48.09)	47.27 (41.40,53.22)	46.69 (40.78,52.69)	41.02 (35.11,47.20)	43.83 (37.83,50.02)	
Marital status (%)						0.031
Living with partner	63.46 (60.80,66.03)	59.18 (53.72,64.42)	67.21 (62.22,71.84)	67.62 (62.89,72.02)	59.69 (53.20,65.86)	
Not living with partner	36.54 (33.97,39.20)	40.82 (35.58,46.28)	32.79 (28.16,37.78)	32.38 (27.98,37.11)	40.31 (34.14,46.80)	
Smoker (%)						0.165
Current	25.51 (23.02,28.17)	22.04 (17.32,27.62)	20.91 (16.50,26.13)	22.11 (17.94,26.92)	23.60 (19.33,28.48)	
Former	38.55 (35.85,41.33)	40.73 (35.48,46.19)	49.50 (44.23,54.78)	49.69 (44.47,54.92)	46.85 (40.95,52.84)	
Never	27.80 (24.86,30.95)	37.23 (31.57,43.26)	29.59 (25.31,34.27)	28.20 (23.38,33.58)	29.55 (24.67,34.94)	
Drinking status (%)	8.13 (6.43,10.23)					0.311
Yes	57.06 (54.02,60.05)	60.65 (54.41,66.57)	53.40 (47.42,59.29)	55.93 (50.22,61.48)	58.27 (53.09,63.27)	
No	42.94 (39.95,45.98)	39.35 (33.43,45.59)	46.60 (40.71,52.58)	44.07 (38.52,49.78)	41.73 (36.73,46.91)	
Physical activity (%)						0.002
Yes	56.22 (53.45,58.95)	61.26 (55.73,66.51)	60.06 (55.52,64.44)	55.59 (50.27,60.78)	48.14 (42.48,53.85)	
No	43.78 (41.05,46.55)	38.74 (33.49,44.27)	39.94 (35.56,44.48)	44.41 (39.22,49.73)	51.86 (46.15,57.52)	
PIR						0.011
Low (<1.30)	25.51 (23.02,28.17)	23.89 (19.47,28.95)	23.20 (19.09,27.90)	25.05 (20.39,30.36)	29.85 (25.70,34.37)	
Medium (1.30-3.5)	38.55 (35.85,41.33)	33.17 (28.18,38.57)	43.14 (37.32,49.16)	39.57 (33.48,46.00)	38.32 (33.24,43.66)	
High (≥3.5)	27.80 (24.86,30.95)	32.82 (26.55,39.76)	28.98 (23.58,35.05)	24.75 (19.52,30.84)	24.85 (20.35,29.98)	
Lack of information	8.13 (6.43,10.23)	10.13 (6.82,14.78)	4.67 (3.09,6.99)	10.64 (7.35,15.16)	6.98 (4.76,10.12)	
Waist circumference (cm)	104.78 (103.91,105.65)	89.84 (88.64,91.04)	100.73 (99.69,101.77)	108.17 (107.11,109.23)	119.96 (118.36,121.57)	<0.001
BMI (kg/m^2^)	29.95 (29.60,30.31)	23.51 (23.14,23.88)	27.72 (27.35,28.08)	31.03 (30.63,31.43)	37.37 (36.73,38.01)	<0.001
SBP (mmHg)	130.44 (129.31,131.57)	132.54 (129.79,135.28)	130.35 (128.08,132.63)	129.14 (127.06,131.22)	129.80 (127.78,131.81)	0.288
DBP (mmHg)	69.59 (68.46,70.72)	68.09 (65.63,70.55)	67.33 (65.54,69.13)	69.50 (67.98,71.02)	73.37 (71.69,75.04)	<0.001
Fasting glucose (mg/dL)	118.76 (116.51,121.00)	105.41 (102.67,108.15)	112.13 (108.75,115.51)	119.10 (114.97,123.22)	138.03 (131.79,144.28)	<0.001
Fasting insulin (μU/mL)	15.17 (14.30,16.04)	7.81 (7.26,8.37)	11.79 (10.77,12.81)	17.92 (15.36,20.49)	22.89 (21.25,24.54)	<0.001
HDL-c (mg/dL)	50.75 (49.65,51.85)	63.74 (61.73,65.75)	50.86 (49.61,52.12)	47.15 (45.51,48.78)	41.56 (40.12,43.00)	<0.001
LDL-c (mg/dL)	105.72 (103.50,107.93)	105.34 (101.36,109.31)	106.72 (102.46,110.98)	104.69 (99.43,109.95)	106.16 (102.19,110.13)	0.952
TG (mg/dL)	150.44 (143.92,156.96)	105.83 (99.24,112.43)	129.42 (122.94,135.90)	154.91 (144.39,165.44)	210.32 (192.34,228.30)	<0.001
TC (mg/dL)	186.11 (183.43,188.80)	190.06 (185.13,194.99)	183.72 (178.88,188.55)	182.05 (175.75,188.35)	188.77 (183.72,193.81)	0.071
HbA1c (%)	6.07 (6.00,6.14)	7.81 (7.26,8.37)	11.79 (10.77,12.81)	17.92 (15.36,20.49)	22.89 (21.25,24.54)	<0.001
eGFR (mL/min/1.73 m^2^)	74.82 (73.59,76.05)	72.84 (70.36,75.33)	74.46 (71.69,77.23)	74.09 (71.45,76.72)	77.88 (75.66,80.11)	0.016
Serum uric acid (mg/dL)	5.96 (5.86,6.06)	5.51 (5.33,5.69)	5.92 (5.75,6.09)	6.16 (5.98,6.35)	6.23 (6.03,6.43)	<0.001
Hypertension (%)						<0.001
Yes	74.65 (71.44,77.61)	66.30 (60.42,71.72)	72.62 (66.34,78.11)	78.94 (74.36,82.89)	80.43 (74.42,85.32)	
No	25.35 (22.39,28.56)	33.70 (28.28,39.58)	27.38 (21.89,33.66)	21.06 (17.11,25.64)	19.57 (14.68,25.58)	
Diabetes (%)						<0.001
Yes	34.87 (32.35,37.48)	16.48 (12.89,20.83)	26.71 (22.04,31.95)	38.89 (34.09,43.92)	56.83 (51.71,61.81)	
No	65.13 (62.52,67.65)	83.52 (79.17,87.11)	73.29 (68.05,77.96)	61.11 (56.08,65.91)	43.17 (38.19,48.29)	
Heart failure (%)						0.075
Yes	26.97 (24.33,29.77)	23.02 (18.36,28.45)	26.06 (21.90,30.70)	26.23 (21.21,31.95)	32.50 (26.80,38.76)	
No	73.03 (70.23,75.67)	76.98 (71.55,81.64)	73.94 (69.30,78.10)	73.77 (68.05,78.79)	67.50 (61.24,73.20)	
CAD (%)						0.100
Yes	71.07 (68.50,73.51)	66.52 (61.16,71.49)	73.23 (68.30,77.64)	73.99 (69.19,78.27)	70.43 (65.79,74.68)	
No	28.93 (26.49,31.50)	33.48 (28.51,38.84)	26.77 (22.36,31.70)	26.01 (21.73,30.81)	29.57 (25.32,34.21)	
Angina (%)						0.231
Yes	28.97 (26.20,31.91)	26.05 (21.30,31.45)	26.73 (21.95,32.13)	30.40 (25.34,35.97)	32.58 (27.34,38.30)	
No	71.03 (68.09,73.80)	73.95 (68.55,78.70)	73.27 (67.87,78.05)	69.60 (64.03,74.66)	67.42 (61.70,72.66)	
Heart attack (%)						0.989
Yes	41.76 (38.98,44.59)	41.33 (36.29,46.56)	41.28 (35.85,46.92)	42.01 (36.97,47.23)	42.39 (36.91,48.08)	
No	58.24 (55.41,61.02)	58.67 (53.44,63.71)	58.72 (53.08,64.15)	57.99 (52.77,63.03)	57.61 (51.92,63.09)	
Stroke (%)						0.049
Yes	32.15 (29.61,34.80)	37.79 (32.15,43.79)	29.46 (24.85,34.53)	28.43 (23.75,33.63)	33.06 (28.25,38.25)	
No	67.85 (65.20,70.39)	62.21 (56.21,67.85)	70.54 (65.47,75.15)	71.57 (66.37,76.25)	66.94 (61.75,71.75)	
Smoking (%)						0.165
Current	22.17 (19.77,24.78)	22.04 (17.32,27.62)	20.91 (16.50,26.13)	22.11 (17.94,26.92)	23.60 (19.33,28.48)	
Former	46.72 (44.10,49.36)	40.73 (35.48,46.19)	49.50 (44.23,54.78)	49.69 (44.47,54.92)	46.85 (40.95,52.84)	
Never	31.11 (28.51,33.83)	37.23 (31.57,43.26)	29.59 (25.31,34.27)	28.20 (23.38,33.58)	29.55 (24.67,34.94)	
Cancer (%)						0.036
Yes	16.45 (14.48,18.62)	19.75 (15.49,24.84)	13.81 (10.70,17.65)	18.40 (15.01,22.35)	13.76 (10.85,17.30)	
No	83.55 (81.38,85.52)	80.25 (75.16,84.51)	86.19 (82.35,89.30)	81.60 (77.65,84.99)	86.24 (82.70,89.15)	

a Data were summarized as mean ± SD or frequency (percentage) according to their data type.METS-IR, metabolic score for insulin resistance; PIR, family poverty income ratio; BMI, body mass index; SBP, systolic blood pressure; DBP, diastolic blood pressure; HDL-c, high-density lipoprotein cholesterol; LDL-c, low-density lipoprotein cholesterol; TG, triglycerides; TC, total cholesterol; HbA1c, hemoglobin A1c; eGFR, estimated glomerular filtration rate.

### 3.2 Association of METS-IR with mortality

The proportional hazards assumption was assessed using the Schoenfeld residuals test. The test results indicated that the assumption holds for all covariates included in the model (p > 0.05 for all variables), suggesting that the hazard ratios for these variables remain constant over time. **Table 2** shows the number of deaths throughout the follow-up period: 1090 from all causes, and 447 from cardiovascular-related causes. Variables selected for inclusion in the multivariable regression models were based on their relevance to the study outcomes, changes in model coefficients, and clinical significance. Specifically, covariates that resulted in a greater than 10% change in the coefficients of the basic model, as well as those supported by prior literature, were included in the Model 3. According to the results of a multivariate Cox proportional hazard regression analysis, in continuous models, after adjusting for all covariates in Model 3, there was no significant association between lnMETS-IR and the risk of all-cause mortality and cardiovascular mortality, with HRs of 0.97 (0.63, 1.50) and 1.13 (0.57, 2.24). In categorical models, the HRs and 95% CIs for all-cause mortality were 1.00 (reference) for Q1, 0.76 (0.64, 0.91) for Q2, 0.82 (0.67, 1.00) for Q3, and 0.98 (0.75, 1.27) for Q4, with no significant trend (P for trend = 0.715). For cardiovascular mortality, the HRs and 95% CIs were 1.00 (reference) for Q1, 0.75 (0.57, 0.99) for Q2, 0.87 (0.63, 1.19) for Q3, and 1.13 (0.76, 1.69) for Q4, with no significant trend (P for trend = 0.662).

**Table 2:** Multivariate Cox regression analysis of METS-IR with all-cause and cardiovascular mortality among participants with CVD.

	Number of deaths	Model 1		Model 2		Model 3	
		HR (95%CI)	P value	HR (95%CI)	P value	HR (95%CI)	P value
All-cause mortality							
lnMETS-IR (per 1 unit increment)	1425	0.57 (0.45, 0.72)	<0.001	0.97 (0.75, 1.26)	0.846	0.97 (0.63, 1.50)	0.888
lnMETS-IR quartile							
Q1	304	1		1		1	
Q2	277	0.75 (0.64, 0.89)	0.001	0.77 (0.65, 0.90)	0.002	0.76 (0.64, 0.91)	0.003
Q3	271	0.74 (0.63, 0.87)	<0.001	0.82 (0.70, 0.97)	0.021	0.82 (0.67, 1.00)	0.048
Q4	238	0.69 (0.58, 0.82)	<0.001	0.99 (0.83, 1.17)	0.869	0.98 (0.75, 1.27)	0.861
P for trend			<0.001		0.882		0.715
Cardiovascular mortality							
lnMETS-IR (per 1 unit increment)	447	0.63 (0.44, 0.92)	0.016	1.20 (0.80, 1.81)	0.374	1.13 (0.57, 2.24)	0.735
lnMETS-IR quartile							
Q1	121	1		1		1	
Q2	109	0.74 (0.57, 0.96)	0.023	0.75 (0.58, 0.98)	0.033	0.75 (0.57, 0.99)	0.043
Q3	112	0.77 (0.59, 0.99)	0.044	0.86 (0.66, 1.12)	0.268	0.87 (0.63, 1.19)	0.372
Q4	105	0.76 (0.59, 0.99)	0.041	1.15 (0.88, 1.50)	0.320	1.13 (0.76, 1.69)	0.545
P for trend			0.116		0.963		0.662

Model 1: no covariates were adjusted.

Model 2: adjusted for age, gender, race.

Model 3: adjusted for covariates in Model 2 plus education level, smoking status, married status, alcohol drinking, BMI, waist circumference, PIR, PA, LDL-c, TC, HbA1c, eGFR, hypertension, diabetes status.

METS-IR, metabolic score for insulin resistance; BMI, body mass index; PIR, family poverty income ratio; PA, physical activity; LDL-c, low-density lipoprotein cholesterol; TC, total cholesterol, HbA1c, hemoglobin A1c; eGFR, estimated glomerular filtration rate; HR, hazard ratio; CI, conﬁdence interval.

**Figure 2:**
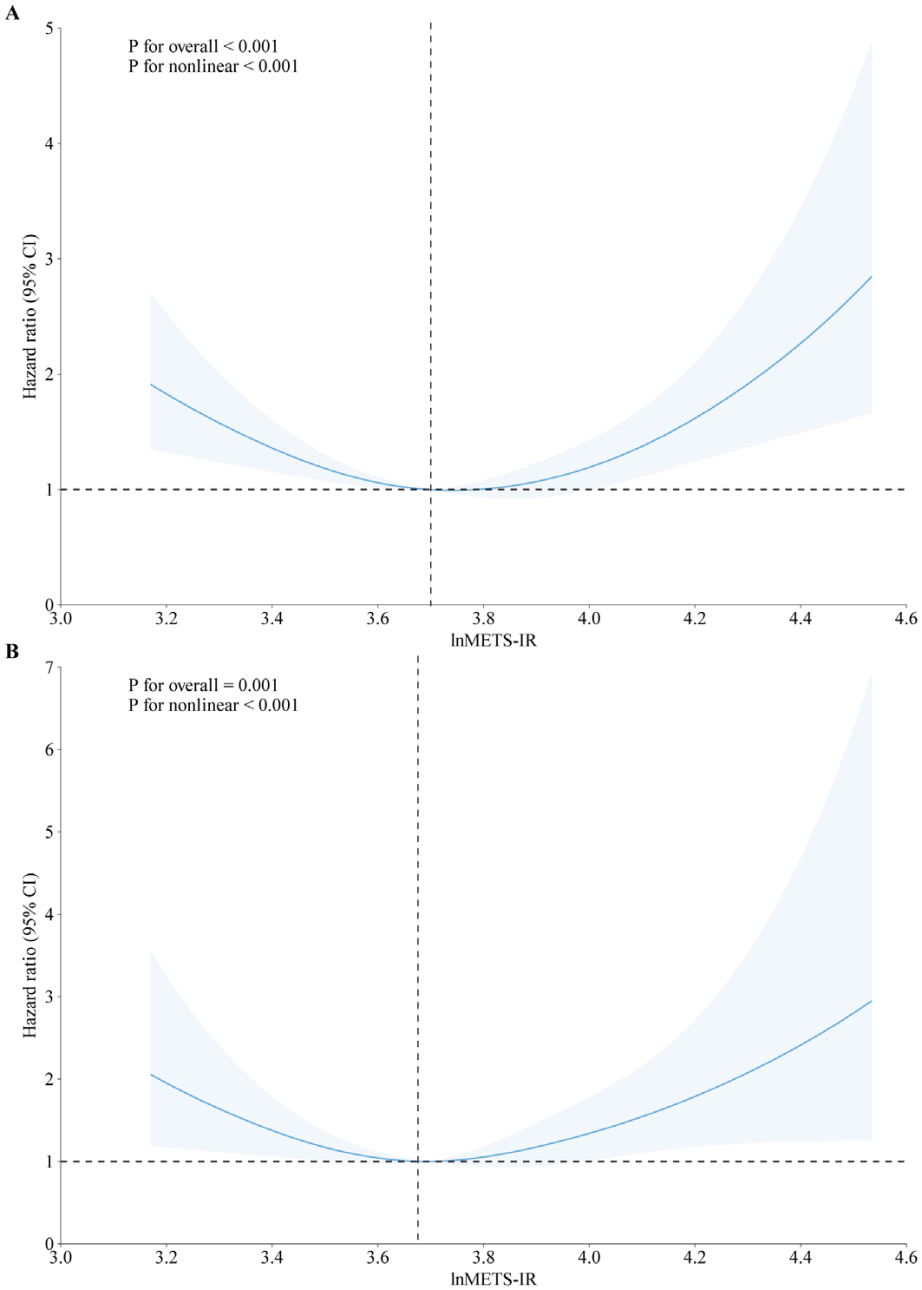
Association between METS-IR and all-cause **(A)** and cardiovascular mortality **(B)** among CVD patients in fully adjusted model. The solid line and red area represent the estimated values and their corresponding 95% CI. HR hazard ratio, CI confidence interval, METS-IR metabolic score for insulin resistance.

Cox proportional hazards regression models with restricted cubic splines (RCS) and smooth curve fitting using the penalized spline approach were employed. It is interesting to note that U-shaped relationships were found between lnMETS-IR and all-cause mortality (P for non-linear < 0.001, **Figure 2A**) and cardiovascular mortality (P for non-linear = 0.003, **Figure 2B**). Segmented Cox regression analysis revealed inflection points for lnMETS-IR in correlation with all-cause and cardiovascular mortality at 3.70 and 3.67, respectively, suggesting that METS-IR exerts a bidirectional effect within specific ranges (**Table 3**). In the lower range of the inflection points, METS-IR was found to be in negative correlation with both outcomes. The risk of all-cause mortality was observed to decline by 75% with each one-unit increase in lnMETS-IR (HR: 0.25, 95% CI: 0.14–0.46, P < 0.001), and a 79% reduction in the risk of cardiovascular mortality was found with each one-unit increase in lnMETS-IR (HR: 0.21, 95% CI: 0.07–0.56, P < 0.001). Conversely, above the inflection points, METS-IR exhibited a positive significant correlation with both outcomes. Each one-unit increase in lnMETS-IR was correlated with a 1.80 elevation in all-cause mortality (HR: 2.80, 95% CI: 1.61–4.88, P < 0.001) and a 2.33 elevation in cardiovascular mortality (HR: 3.33, 95% CI: 1.43–7.75, P = 0.005).

**Table 3:** Threshold effect analysis of METS-IR and mortality.

	Adjusted HR (95% CI)	P-value
All-cause mortality		
Total	0.97 (0.63, 1.50)	0.888
Fitting by two-piecewise Cox proportional risk model		
Inflection point	3.70	
lnMETS-IR < 3.70	0.25 (0.14, 0.46)	<0.001
lnMETS-IR ≥ 3.70	2.80 (1.61, 4.88)	<0.001
P for Log-likelihood ratio	<0.001	
Cardiovascular mortality		
Total	1.13 (0.57, 2.24)	0.735
Fitting by two-piecewise Cox proportional risk model		
Inflection point	3.67	
lnMETS-IR < 3.67	0.21 (0.07, 0.56)	0.002
lnMETS-IR ≥ 3.67	3.33 (1.43, 7.75)	0.005
P for Log-likelihood ratio	<0.001	

Cox proportional hazard models were used to estimate HR and 95% CI. Adjusted for age, gender, race, education level, education level, smoking status, married status, alcohol drinking, BMI, waist circumference, PIR, PA, LDL-c, TC, HbA1c, eGFR, hypertension, diabetes status.

METS-IR, metabolic score for insulin resistance; BMI, body mass index; PIR, family poverty income ratio; PA, physical activity; LDL-c, low-density lipoprotein cholesterol; TC, total cholesterol, HbA1c, hemoglobin A1c; eGFR, estimated glomerular filtration rate; HR, hazard ratio; CI, conﬁdence interval.

**Figure 3:**
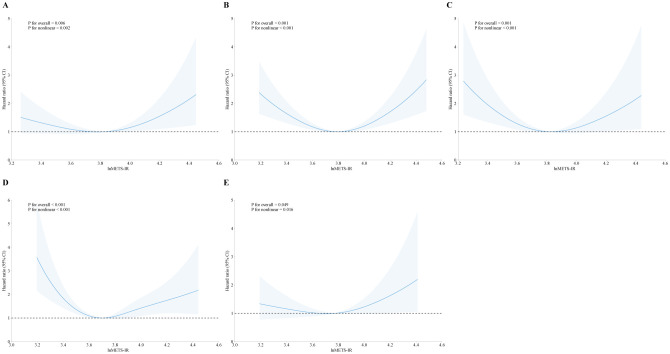
The association of METS-IR with all-cause mortality among patients with heart failure **(A)**, coronary heart disease **(B)**, heart attack **(C)**, angina **(D)**, and stroke **(E)**.

**Figure 4:**
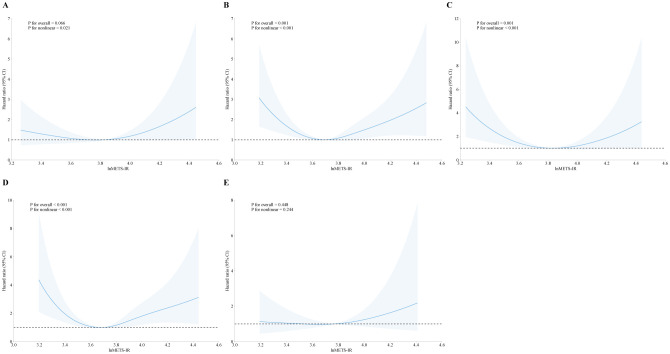
The association of METS-IR with cardiovascular mortality among patients with heart failure **(A)**, coronary heart disease **(B)**, heart attack **(C)**, angina **(D)**, and stroke **(E)**.

In addition, we explored the correlation between METS-IR and mortality stratified by heart failure, coronary heart disease, heart attack, angina, and stroke, as showed in **Figures 3**, **4**. METS-IR exhibited an L-shaped or U-shaped association with all-cause mortality among these five groups (**Figure 3**). Similarly, an L-shaped or U-shaped association with all-cause mortality was also found among heart failure, coronary heart disease, heart attack, and angina patients, while no significant nonlinear association was observed among stroke patients (P for nonlinear = 0.244) (**Figure 4**).

### 3.3 Stratified analyses

**Figure 5:**
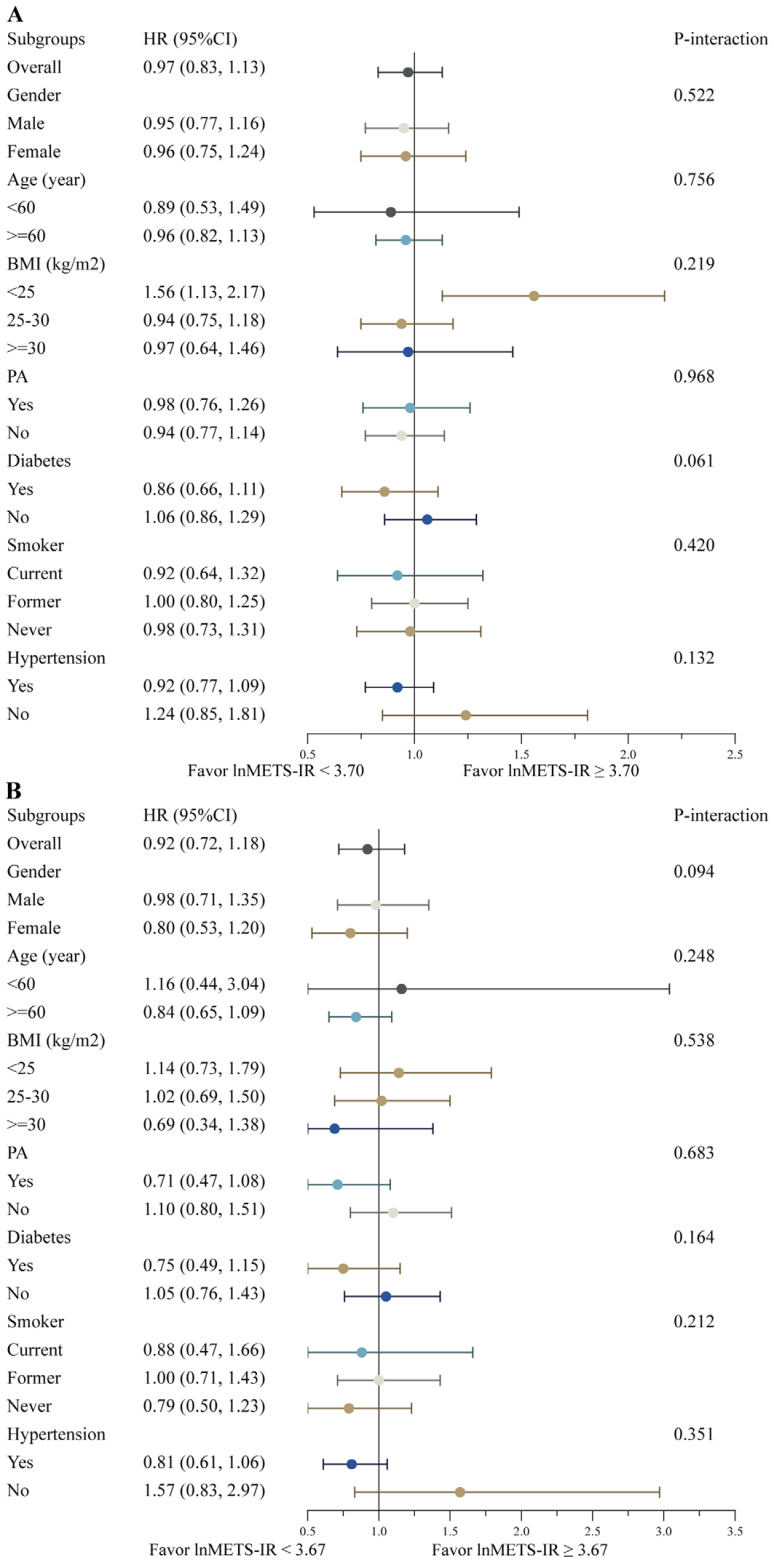
Stratified analyses of the associations between the METS-IR and all-cause **(A)** and cardiovascular mortality **(B)** among individuals with CVD. Hazard ratios were estimated using a two-piecewise Cox proportional risk model on both sides of the inflection point (all-cause mortality: 3.70; cardiovascular mortality: 3.67) and adjusted for confounders.

The survival advantage correlated with a higher lnMETS-IR (≥ 3.70 for all-cause mortality and ≥ 3.67 for cardiovascular mortality) compared to a lower lnMETS-IR (< 3.70 for all-cause mortality and < 3.67 for cardiovascular mortality) in CVD individuals remained consistent across various subgroups stratified by age, gender, BMI, PA, diabetes, smoking status, and hypertension (all P for interaction > 0.05), as shown in **Figure 5**. No significant interaction was identified between METS-IR and any of the stratified variables.

### 3.4 Sensitivity analysis

In the sensitivity analyses, after excluding individuals with incomplete baseline covariate data, a total of 2129 participants were included. The results indicated that the relationships between METS-IR and both all-cause and cardiovascular mortality were stable (see **Supplementary Table S2**). Furthermore, after the exclusion of patients who declared a cancer diagnosis at the outset of the study, the remaining cohort contained 2112 individuals. After adjustment for all confounders, the associations between METS-IR and mortality remained consistent (see **Supplementary Table S3**).

## 4 Discussion

To the best of our knowledge, this is the first study to reveal a U-shaped relationship between lnMETS-IR and the risks of all-cause and cardiovascular mortality in patients with CVD, with thresholds identified at 3.70 and 3.67. Interaction and sensitivity analyses indicated that these findings are robust. These findings suggest that METS-IR may be a useful predictor of mortality in patients with CVD. Further studies are needed to validate its clinical applicability.

METS-IR is an emerging biomarker with numerous advantages in clinical practice. Unlike the HEC method, the calculation of METS-IR requires only the measurement of fasting blood glucose, lipid profiles, height, and weight. Therefore, METS-IR serves as an easily accessible, cost-effective, and practical method for evaluating IR in patients with CVD. Since its introduction, several studies have highlighted the potential of METS-IR as a robust predictor of metabolic and cardiovascular outcomes. For instance, in Japan, METS-IR was found to independently predict future type 2 diabetes in non-obese adults (27). In Korea, METS-IR showed a stronger predictive value for ischemic heart disease (IHD) in non-diabetic individuals compared to metabolic syndrome (MetS) (16). Moreover, METS-IR was positively associated with the risk of cardiovascular events in hypertensive patients (28, 29) and demonstrated a “J-shaped” relationship with stroke and ischemic stroke risk in individuals with hypertension and obstructive sleep apnea (30). Notably, a study by Zhou et al. in heart failure patients showed that higher METS-IR levels were associated with increased mortality risk, with adjusted hazard ratios for all-cause and cardiovascular deaths being 2.48 (95% CI: 2.10–2.93) and 2.29 (95% CI: 1.83–2.87), respectively (26). METS-IR exhibits a nonlinear “U-shaped” relationship with mortality in the general population, with stronger associations than other insulin resistance markers like TyG index, TG/HDL-c, and HOMA-IR (19). These findings suggest that METS-IR may have unique prognostic value across different populations, particularly in those with hypertension, heart failure, or diabetes, underscoring the need for further studies to explore its utility in diverse patient groups. Nevertheless, no research has examined the relationship between METS-IR and the mortality risk in CVD patients. Comparison with previous studies on METS-IR and cardiovascular outcomes, our study reaches conclusions consistent with prior findings. Additionally, we extend this work by emphasizing the prognostic value of METS-IR in a broader CVD population. This study provides the first demonstration of a U-shaped relationship between lnMETS-IR and both all-cause and cardiovascular mortality. Our analysis confirmed that the proportional hazards assumption was met for all covariates (p > 0.05), supporting the validity of the Cox proportional hazards model for our data. We also analyze specific CVD subtypes, including heart failure, coronary heart disease, heart attack, and angina. Furthermore, through subgroup analyses based on different BMI categories, we highlight the broader utility of METS-IR in predicting metabolic and cardiovascular risks.

In agreement with previous studies (31, 32), the results of this study demonstrated a U-shaped relationship between METS-IR and mortality in patients with CVD. Notably, the inflection point for all-cause mortality was approximately 3.70, while the inflection point for cardiovascular mortality was approximately 3.67. This identification of threshold values enables physicians to focus on specific patient subgroups for closer monitoring and timely intervention. In addition, we further explored the relationship of METS-IR in specific CVD subgroups and confirmed that the effect of METS-IR on mortality was consistent across CVD subgroups, showing either a J- or U-shaped relationship, except for cardiovascular mortality in the stroke population, which did not show a significant nonlinear relationship. We consider that the inconsistency of this result may be due to the small sample size of the included stroke population.

An increasing number of studies indicate that metabolic factors may be associated with a worse prognosis in patients with CVD. The impact of BMI on the prognosis of patients with CVD remains a topic of active debate in the scientific community. Studies have demonstrated a J- or U-shaped correlation between BMI and the risk of developing cardiovascular events or all-cause mortality (33, 34). Similarly, low BMI is found to be associated with elevated levels of BNP and N-terminal pro-B-type natriuretic peptide in patients with chronic heart failure, which may result in a poorer prognosis, particularly when chronic heart failure progresses to cardiac cachexia, which can further impact survival outcomes (35). In the subgroup analysis, we observed that when the lnMETS-IR value exceeded a threshold, its impact on mortality appeared to be more pronounced in individuals with a BMI < 25. We hypothesize that obese patients may manifest symptoms earlier, leading to timely intervention and optimal pharmacotherapy, while benefiting from the metaboloprotective effects of adiposity and enhanced metabolic reserves. Conversely, weight loss in individuals at elevated cardiovascular risk may be attributed to cachexia associated with severe comorbidities, such as malignancies or chronic infections (29).

This finding likely reflects the complex interplay between metabolic function and mortality risk. When METS-IR is low, despite mild IR, the mortality risk remains elevated, particularly in low-BMI individuals, who typically have lower metabolic reserves and are more vulnerable to acute illnesses or chronic metabolic disturbances (36). In contrast, high METS-IR values are generally associated with increased IR, particularly in obese individuals. The accumulation of visceral fat not only exacerbates IR (37) but also promotes chronic low-grade inflammation through the secretion of pro-inflammatory cytokines such as TNF-α and IL-6 (38), which further increases the risk of cardiovascular events and endothelial dysfunction, ultimately elevating mortality risk.

It is noteworthy that there is a correlation between lower IR levels and lower fasting blood glucose levels (39, 40). Studies have demonstrated that episodes of hypoglycemia and rapid fluctuations in blood glucose levels lead to an increase in the levels of counter-regulatory hormones, including epinephrine and norepinephrine. These hormones induce platelet aggregation and vasoconstriction, which may accelerate ischemia in the cardiovascular system (41). Extremely low triglyceride and fasting glucose levels could indicate inadequate nutritional status. A J-shaped relationship has been identified between blood glucose levels and cardiovascular events or all-cause mortality. Specifically, it has been revealed that the lower the fasting blood glucose level, the worse the prognosis (42). A study conducted in Korea demonstrated that severe hypoglycemia was associated with an elevated risk of cardiovascular events and all-cause mortality in patients with T2DM (43). Similarly, decreased TG levels were found to be associated with a higher risk of developing hemorrhagic stroke among women, and had been found to serve as a predictor of cardiac death in heart failure patients (44). We discussed the potential mechanisms behind this U-shaped relationship. Significantly, the METS-IR provides valuable information on lipid metabolism abnormalities, as well as serves as an indicator of oxidative stress levels and cardiovascular risk that surpasses conventional lipid assessments. This enhanced capability may lead to a more comprehensive understanding of cardiometabolic risk factors associated with CVD outcomes. Our findings are consistent with previous studies, which also observed a U-shaped (19) or J-shaped (31) relationship between METS-IR and mortality. We believe this U-shaped relationship has important clinical implications and suggests that when evaluating metabolic health, it is crucial to consider the full spectrum of metabolic states, rather than focusing solely on absolute METS-IR values. Thus, METS-IR could be a useful tool for assessing IR and predicting outcomes in individuals with CVD.

In addition to the direct link between METS-IR and mortality, clinical phenotypes such as coronary artery calcification (45), subclinical myocardial injury (46), and other cardiovascular risks (47) may mediate this relationship. METS-IR is associated with increased arterial stiffness, subclinical atherosclerosis, and endothelial dysfunction (48), all of which are key factors in CVD progression and mortality risk. These clinical manifestations highlight the importance of considering not only metabolic markers but also cardiovascular pathophysiology when evaluating mortality risk in CVD patients. Future studies should investigate the role of these phenotypes in mediating the METS-IR mortality association.

The exact mechanism by which METS-IR leads to increased mortality remains unclear. IR contributes to CVD mainly by disrupting glucose and lipid metabolism, increasing vascular stiffness and endothelial dysfunction, and inducing oxidative stress and inflammatory responses. Firstly, the migration of smooth muscle cells and deposition of collagen at sites of damaged endothelial cells are thought to be mediated by elevated levels of oxidative stress associated with IR. Secondly, insulin-mediated nitric oxide (NO) production is conducive to circulation and glucose utilization. A reduction in nitric oxide production due to IR in the endothelium serves to further impair endothelial function and increase damage to endothelial cells (48, 49). Thirdly, insulin resistance reduces insulin sensitivity in the liver and muscle tissue, thereby impeding insulin utilization and glucose uptake (50). This condition leads to hyperglycemia, exacerbation of the local inflammatory response, smooth muscle cell proliferation, collagen deposition and ultimately vascular ageing and hardening (51). Fourthly, IR has the potential to trigger thrombosis and activate platelets through mechanisms such as upregulation of the expression of adhesion-inducing factor and thromboxane A2-dependent tissue factor and activation of fibrinogen activator inhibitor-1 (52, 53). In combination, these physiological processes contribute to the onset and progression of CVD, ultimately resulting in poor clinical outcomes.

METS-IR has significant potential for clinical risk stratification and personalized interventions, especially for patients at high risk of metabolic diseases, cardiovascular conditions, and type 2 diabetes. By identifying individuals at greater metabolic risk, it can guide clinicians in tailoring preventive measures and therapies, such as lifestyle modifications and early pharmacological treatments. METS-IR could also be integrated into routine clinical screenings to identify high-risk individuals early, allowing for timely intervention. Future research should focus on validating METS-IR across diverse populations and exploring its integration into digital health tools to improve accessibility and clinical utility. However, there are several limitations that should be considered. First, as a cross-sectional study, we do not establish a causal relationship between METS-IR and mortality, and larger-scale studies are needed to validate these results. Second, the potential impact of fluctuations in METS-IR over time on its correlation with mortality remains unclear due to the lack of continuous monitoring of the METS-IR in our study. Third, residual confounders, such as long-term lifestyle habits, dietary patterns, genetic susceptibility, and environmental factors, may still influence the results. For example, individuals’ eating habits, physical activity levels, and other health behaviors may change over time, significantly impacting their metabolic status and contributing to variations in METS-IR. Future research could expand on these potential confounders by incorporating genetic data, environmental exposures, and more comprehensive behavioral assessments. Additionally, longitudinal study designs could more closely track participants’ lifestyle factors and analyze their cumulative effects over time. Moreover, the presence of CVD was self-reported and certain lifestyle variables were based on questionnaires, which introduced the possibility of recall bias or inaccuracies in the data. Future studies should incorporate more objective measures to reduce this bias.

## 5 Conclusions

Our findings show that METS-IR is a valuable tool for predicting all-cause and cardiovascular mortality in CVD patients. Particularly noteworthy is the presence of a U-shaped relationship between the METS-IR and both all-cause and CVD mortality. Furthermore, the identified threshold could serve as a target for interventions aimed at reducing the risk of premature mortality. Further research is required to investigate whether interventions targeting METS-IR can result in better clinical outcomes for individuals in the future.

## Data Availability

Publicly available datasets were analyzed in this study. This data can be found here: https://www.cdc.gov/nchs/nhanes/index.htm.

## Supplementary material

Supplementary Figure 1 Summary of Schoenfeld Residual Plots for covariates on all-cause mortality.

Supplementary Figure 2 Summary of Schoenfeld Residual Plots for covariates on cardiovascular mortality.

## References

[1] Martin SS Aday AW Almarzooq ZI Anderson CAM Arora P Avery CL. 2024 heart disease and stroke statistics: A report of US and global data from the American heart association. Circulation. (2024) 149:e347–913. doi: 10.1161/CIR.0000000000001209 PMC12146881 38264914

[2] Roth GA Mensah GA Johnson CO Addolorato G Ammirati E Baddour LM. global burden of cardiovascular diseases and risk factors, 1990-2019: update from the GBD 2019 study. J Am Coll Cardiol. (2020) 76:2982–3021. doi: 10.1016/j.jacc.2020.11.010 33309175 PMC7755038

[3] Mozaffarian D Benjamin EJ Go AS Arnett DK Blaha MJ Cushman M. heart disease and stroke statistics-2016 update: A report from the American heart association. Circulation. (2016) 133:e38–360. doi: 10.1161/CIR.0000000000000350 26673558

[4] Nilsson PM Tuomilehto J Rydén L. The metabolic syndrome - What is it and how should it be managed? Eur J Prev Cardiol. (2019) 26(2_suppl):33–46. doi: 10.1177/2047487319886404 31766917

[5] Gurka MJ Guo Y Filipp SL DeBoer MD. Metabolic syndrome severity is significantly associated with future coronary heart disease in Type 2 diabetes. Cardiovasc Diabetol. (2018) 17:17. doi: 10.1186/s12933-017-0647-y 29351794 PMC5775549

[6] Decker JJ Norby FL Rooney MR Soliman EZ Lutsey PL Pankow JS. Metabolic syndrome and risk of ischemic stroke in atrial fibrillation: ARIC study. Stroke. (2019) 50:3045–50. doi: 10.1161/STROKEAHA.119.025376 PMC6817422 31623543

[7] DeBoer MD Gurka MJ Golden SH Musani SK Sims M Vishnu A. Independent associations between metabolic syndrome severity and future coronary heart disease by sex and race. J Am Coll Cardiol. (2017) 69:1204–5. doi: 10.1016/j.jacc.2016.10.088 PMC5481721 28254184

[8] DeFronzo RA Tripathy D. Skeletal muscle insulin resistance is the primary defect in type 2 diabetes. Diabetes Care. (2009) 32 Suppl 2:S157–163. doi: 10.2337/dc09-S302 PMC2811436 19875544

[9] Mauvais-Jarvis F. Sex differences in metabolic homeostasis, diabetes, and obesity. Biol Sex Differ. (2015) 6:14. doi: 10.1186/s13293-015-0033-y 26339468 PMC4559072

[10] Cheng YJ Imperatore G Geiss LS Saydah SH Albright AL Ali MK. Trends and disparities in cardiovascular mortality among U.S. Adults with and without self-reported diabetes, 1988-2015. Diabetes Care. (2018) 41:2306–15. doi: 10.2337/dc18-0831 PMC7849201 30131397

[11] DeFronzo RA Tobin JD Andres R. Glucose clamp technique: a method for quantifying insulin secretion and resistance. Am J Physiol. (1979) 237:E214–223. doi: 10.1152/ajpendo.1979.237.3.E214 382871

[12] Bello-Chavolla OY Almeda-Valdes P Gomez-Velasco D Viveros-Ruiz T Cruz-Bautista I Romo-Romo A. METS-IR, a novel score to evaluate insulin sensitivity, is predictive of visceral adiposity and incident type 2 diabetes. Eur J Endocrinol. (2018) 178:533–44. doi: 10.1530/EJE-17-0883 29535168

[13] Zeng J Zhang T Yang Y Wang J Zheng D Hou Y. Association between a metabolic score for insulin resistance and hypertension: results from National Health and Nutrition Examination Survey 2007-2016 analyses. Front Endocrinol (Lausanne). (2024) 15:1369600. doi: 10.3389/fendo.2024.1369600 38711979 PMC11070536

[14] Seifi N Nosrati M Koochackpoor G Aghasizadeh M Bahari H Namdar HB. The association between hyperuricemia and insulin resistance surrogates, dietary- and lifestyle insulin resistance indices in an Iranian population: MASHAD cohort study. Nutr J. (2024) 23:5. doi: 10.1186/s12937-023-00904-2 38172828 PMC10765631

[15] Pan L Zou H Meng X Li D Li W Chen X. Predictive values of metabolic score for insulin resistance on risk of major adverse cardiovascular events and comparison with other insulin resistance indices among Chinese with and without diabetes mellitus: Results from the 4C cohort study. J Diabetes Investig. (2023) 14:961–72. doi: 10.1111/jdi.14024 PMC10360377 37132055

[16] Yoon J Jung D Lee Y Park B. The Metabolic Score for Insulin Resistance (METS-IR) as a Predictor of Incident Ischemic Heart Disease: A Longitudinal Study among Korean without Diabetes. J Pers Med. (2021) 11(8):742. doi: 10.3390/jpm11080742 34442386 PMC8399912

[17] Wang S Zhang X Keerman M Guo H He J Maimaitijiang R. Impact of the baseline insulin resistance surrogates and their longitudinal trajectories on cardiovascular disease (coronary heart disease and stroke): a prospective cohort study in rural China. Front Endocrinol (Lausanne). (2023) 14:1259062. doi: 10.3389/fendo.2023.1259062 38189050 PMC10767254

[18] Shangguan Q Liu Q Yang R Zhang S Sheng G Kuang M. Predictive value of insulin resistance surrogates for the development of diabetes in individuals with baseline normoglycemia: findings from two independent cohort studies in China and Japan. Diabetol Metab Syndrome. (2024) 16:68. doi: 10.1186/s13098-024-01307-x PMC10943817 38491516

[19] Duan M Zhao X Li S Miao G Bai L Zhang Q. Metabolic score for insulin resistance (METS-IR) predicts all-cause and cardiovascular mortality in the general population: evidence from NHANES 2001–2018. Cardiovasc Diabetol. (2024) 23:243. doi: 10.1186/s12933-024-02334-8 38987779 PMC11238348

[20] Organization WH. International statistical classification of diseases and related health problems: volume 3. Alphabetical index. World Health Organization (1994).

[21] Zou X Zhou X Zhu Z Ji L. Novel subgroups of patients with adult-onset diabetes in Chinese and US populations. Lancet Diabetes Endocrinol. (2019) 7:9–11. doi: 10.1016/S2213-8587(18)30316-4 30577891

[22] Levey AS Stevens LA Schmid CH Zhang YL Castro AF 3rd Feldman HI. A new equation to estimate glomerular filtration rate. Ann Intern Med. (2009) 150:604–12. doi: 10.7326/0003-4819-150-9-200905050-00006 PMC2763564 19414839

[23] Vandenbroucke JP von Elm E Altman DG Gøtzsche PC Mulrow CD Pocock SJ. Strengthening the Reporting of Observational Studies in Epidemiology (STROBE): explanation and elaboration. Int J Surg. (2014) 12:1500–24. doi: 10.1016/j.ijsu.2014.07.014 25046751

[24] Harrell FE. Regression Modeling Strategies. Springer New York (2019). doi: 10.1007/978-1-4757-3462-1

[25] Burnhan KP Anderson DR. Model selection and multi-model inference: a practical Information-theoretic approach. Technometrics. (2002) 45(2):181–1. doi: 10.1198/tech.2003.s146

[26] Zhou Y Xie Y Du L Dong J He K. Metabolic score for insulin resistance as a predictor of mortality in heart failure with preserved ejection fraction: results from a multicenter cohort study. Diabetol Metab Syndr. (2024) 16:220. doi: 10.1186/s13098-024-01463-0 39261964 PMC11389121

[27] Cai XT Zhu Q Liu SS Wang MR Wu T Hong J. Associations between the metabolic score for insulin resistance index and the risk of type 2 diabetes mellitus among non-obese adults: insights from a population-based cohort study. Int J Gen Med. (2021) 14:7729–40. doi: 10.2147/IJGM.S336990 PMC8579827 34785931

[28] Yang W Cai X Hu J Wen W Mulalibieke H Yao X. the metabolic score for insulin resistance (METS-IR) predicts cardiovascular disease and its subtypes in patients with hypertension and obstructive sleep apnea. Clin Epidemiol. (2023) 15:177–89. doi: 10.2147/CLEP.S395938 PMC9939804 36815173

[29] Zhang L Yu C Wang T Zhou W Bao H Cheng X. Association of the metabolic score for insulin resistance with cardiovascular diseases, cardiovascular and all-cause mortality in Chinese hypertensive population. Front Endocrinol (Lausanne). (2023) 14:1326436. doi: 10.3389/fendo.2023.1326436 38523869 PMC10957551

[30] Cai X Hu J Zhu Q Wang M Liu S Dang Y. Relationship of the metabolic score for insulin resistance and the risk of stroke in patients with hypertension: A cohort study. Front Endocrinol (Lausanne). (2022) 13:1049211. doi: 10.3389/fendo.2022.1049211 36545329 PMC9760826

[31] Kityo A Lee SA. Association of cardiometabolic factors and insulin resistance surrogates with mortality in participants from the Korean Genome and Epidemiology Study. Lipids Health Dis. (2023) 22:210. doi: 10.1186/s12944-023-01981-2 38041195 PMC10691157

[32] Wang Z Xie J Wang J Feng W Liu N Liu Y. Association between a novel metabolic score for insulin resistance and mortality in people with diabetes. Front Cardiovasc Med. (2022) 9:895609. doi: 10.3389/fcvm.2022.895609 35647046 PMC9133456

[33] Zheng W McLerran DF Rolland B Zhang X Inoue M Matsuo K. Association between body-mass index and risk of death in more than 1 million Asians. N Engl J Med. (2011) 364:719–29. doi: 10.1056/NEJMoa1010679 PMC4008249 21345101

[34] Berrington de Gonzalez A Hartge P Cerhan JR Flint AJ Hannan L MacInnis RJ. et al: Body-mass index and mortality among 1.46 million white adults. N Engl J Med. (2010) 363:2211–9. doi: 10.1056/NEJMoa1000367 PMC3066051 21121834

[35] Newman MS Nguyen T Watson MJ Hull RW Yu HG. Transcriptome profiling reveals novel BMI- and sex-specific gene expression signatures for human cardiac hypertrophy. Physiol Genomics. (2017) 49:355–67. doi: 10.1152/physiolgenomics.00122.2016 PMC5538878 28500252

[36] Scrimshaw NS SanGiovanni JP. Synergism of nutrition, infection, and immunity: an overview. Am J Clin Nutr. (1997) 66:464s–77s. doi: 10.1093/ajcn/66.2.464S 9250134

[37] Schetz M De Jong A Deane AM Druml W Hemelaar P Pelosi P. Obesity in the critically ill: a narrative review. Intensive Care Med. (2019) 45:757–69. doi: 10.1007/s00134-019-05594-1 30888440

[38] Tchernof A Després JP. Pathophysiology of human visceral obesity: an update. Physiol Rev. (2013) 93:359–404. doi: 10.1152/physrev.00033.2011 23303913

[39] Sarwar N Gao P Seshasai SR Gobin R Kaptoge S Di Angelantonio E. Diabetes mellitus, fasting blood glucose concentration, and risk of vascular disease: a collaborative meta-analysis of 102 prospective studies. Lancet. (2010) 375:2215–22. doi: 10.1016/S0140-6736(10)60484-9 PMC2904878 20609967

[40] Park C Guallar E Linton JA Lee DC Jang Y Son DK. Fasting glucose level and the risk of incident atherosclerotic cardiovascular diseases. Diabetes Care. (2013) 36:1988–93. doi: 10.2337/dc12-1577 PMC3687304 23404299

[41] Galassetti P Davis SN. Effects of insulin per se on neuroendocrine and metabolic counter-regulatory responses to hypoglycaemia. Clin Sci (Lond). (2000) 99:351–62. doi: 10.1042/cs0990351 11052915

[42] Lee JH Han K Huh JH. The sweet spot: fasting glucose, cardiovascular disease, and mortality in older adults with diabetes: a nationwide population-based study. Cardiovasc Diabetol. (2020) 19:44. doi: 10.1186/s12933-020-01021-8 32238157 PMC7110776

[43] Yun JS Park YM Han K Cha SA Ahn YB Ko SH. Severe hypoglycemia and the risk of cardiovascular disease and mortality in type 2 diabetes: a nationwide population-based cohort study. Cardiovasc Diabetol. (2019) 18:103. doi: 10.1186/s12933-019-0909-y 31412855 PMC6694505

[44] Kozdag G Ertas G Emre E Akay Y Celikyurt U Sahin T. Low serum triglyceride levels as predictors of cardiac death in heart failure patients. Tex Heart Inst J. (2013) 40(5):521–8. doi: 10.1097/FJC.0b013e3182a50c45 PMC3853839 24391311

[45] Wang Z Hui X Huang X Li J Liu N. Relationship between a novel non-insulin-based metabolic score for insulin resistance (METS-IR) and coronary artery calcification. BMC Endocr Disord. (2022) 22:274. doi: 10.1186/s12902-022-01180-7 36357872 PMC9647937

[46] Wang Z Li W Li J Liu N. The nonlinear correlation between a novel metabolic score for insulin resistance and subclinical myocardial injury in the general population. Front Endocrinol (Lausanne). (2022) 13:889379. doi: 10.3389/fendo.2022.889379 35685209 PMC9171429

[47] Shulman GI. Ectopic fat in insulin resistance, dyslipidemia, and cardiometabolic disease. N Engl J Med. (2014) 371:2237–8. doi: 10.1056/NEJMra1011035 25470706

[48] Hill MA Yang Y Zhang L Sun Z Jia G Parrish AR. Insulin resistance, cardiovascular stiffening and cardiovascular disease. Metabolism. (2021) 119:154766. doi: 10.1016/j.metabol.2021.154766 33766485

[49] Molina MN Ferder L Manucha W. Emerging role of nitric oxide and heat shock proteins in insulin resistance. Curr Hypertens Rep. (2016) 18:1. doi: 10.1007/s11906-015-0615-4 26694820

[50] Brown AE Walker M. Genetics of insulin resistance and the metabolic syndrome. Curr Cardiol Rep. (2016) 18:75. doi: 10.1007/s11886-016-0755-4 27312935 PMC4911377

[51] Xu H Barnes GT Yang Q Tan G Yang D Chou CJ. Chronic inflammation in fat plays a crucial role in the development of obesity-related insulin resistance. J Clin Invest. (2003) 112:1821–30. doi: 10.1172/JCI200319451 PMC296998 14679177

[52] Iwaki T Urano T Umemura K. PAI-1, progress in understanding the clinical problem and its aetiology. Br J Haematol. (2012) 157:291–8. doi: 10.1111/j.1365-2141.2012.09074.x 22360729

[53] Gerrits AJ Koekman CA van Haeften TW Akkerman JW. Platelet tissue factor synthesis in type 2 diabetic patients is resistant to inhibition by insulin. Diabetes. (2010) 59:1487–95. doi: 10.2337/db09-1008 PMC2874710 20200314

