# Supplementary figures and images for: The association between the metabolic score for insulin resistance and mortality in patients with cardiovascular disease: a national cohort study
Source: Front Endocrinol (Lausanne). 2024 Dec 18;15:1479980. doi: 10.3389/fendo.2024.1479980 (PMC11695433; doi:10.3389/fendo.2024.1479980)

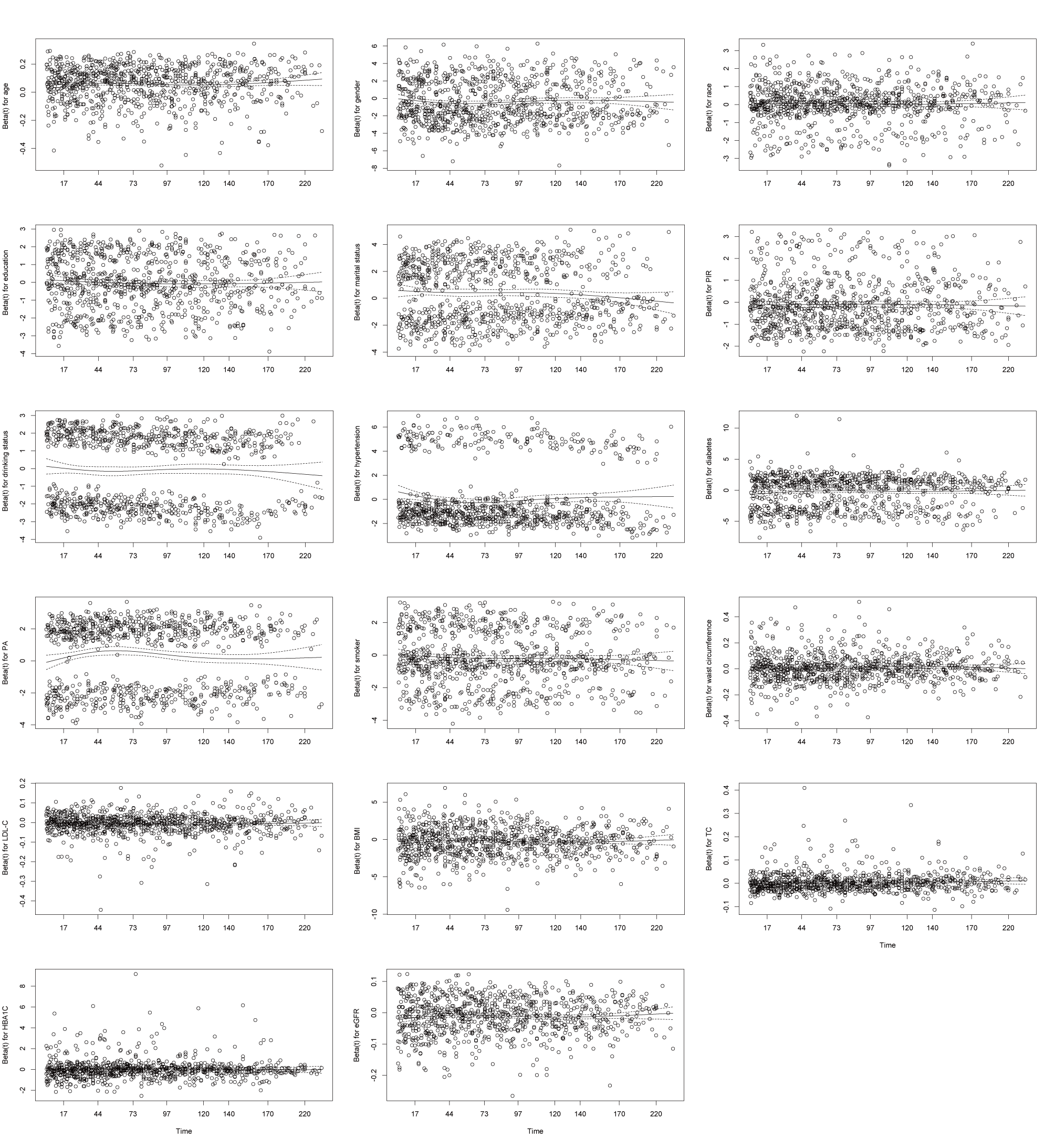

Supplement: Supplementary Figure 1 — Summary of Schoenfeld Residual Plots for covariates on all-cause mortality. [file Image1.tif]

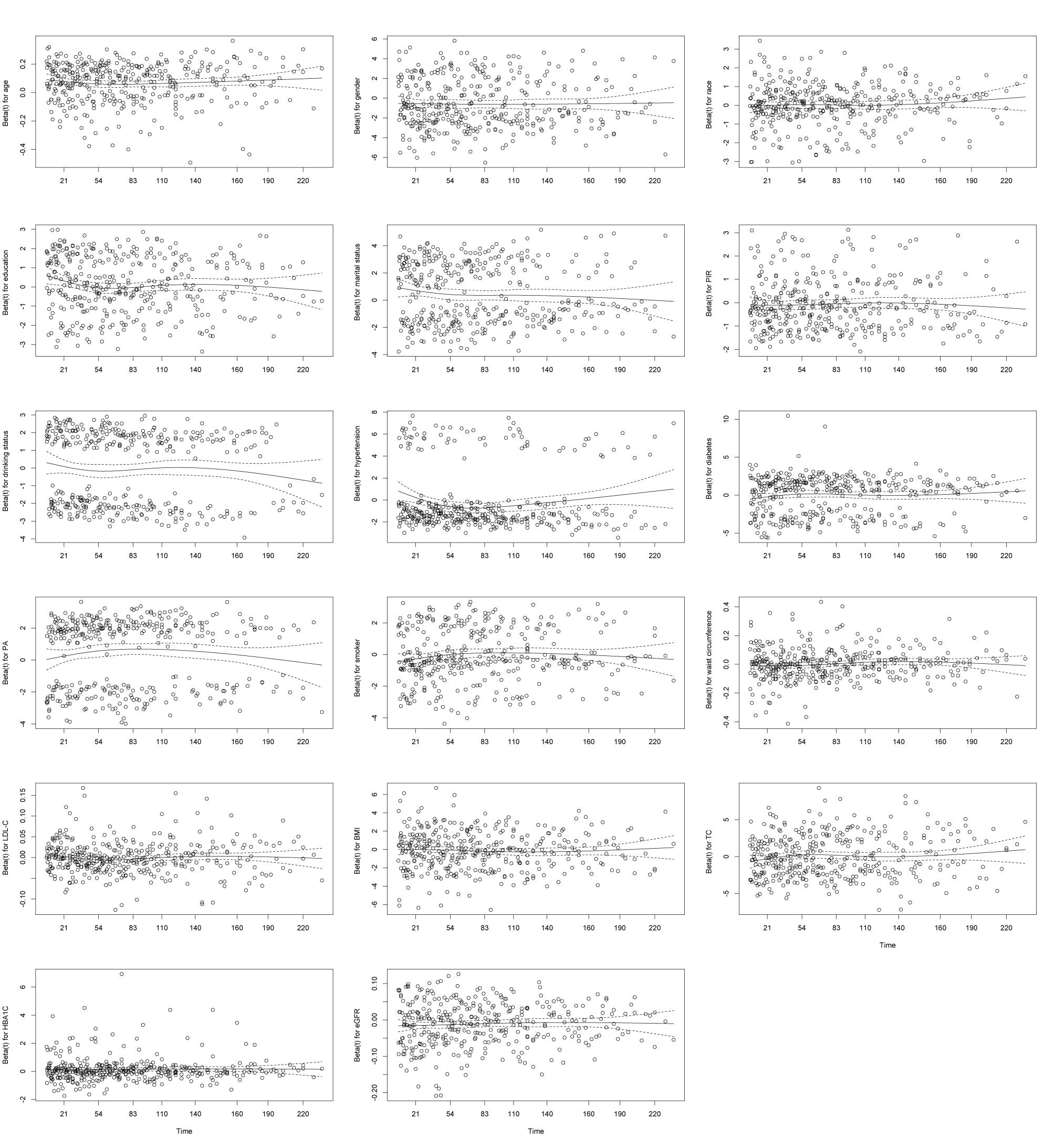

Supplement: Supplementary Figure 2 — Summary of Schoenfeld Residual Plots for covariates on cardiovascular mortality. [file Image2.tif]
