# Supplementary material for: The association between the metabolic score for insulin resistance and mortality in patients with cardiovascular disease: a national cohort study
Source: Front Endocrinol (Lausanne). 2024 Dec 18;15:1479980. doi: 10.3389/fendo.2024.1479980 (PMC11695433; doi:10.3389/fendo.2024.1479980)
Supplement: Supplementary file 3 [file Table1.docx]

**Table S1.** The proportion of missing covariates and imputation methods

| **Variable** | **Proportion of missing (%)** | **Imputation Methods** |
| --- | --- | --- |
| Education | 0.318 | polyreg |
| Married status | 0.557 | polyreg |
| Smoking status | 0.039 | polyreg |
| Alcohol drinking | 7.396 | polyreg |
| Waist circumference | 5.567 | pmm |
| LDL-c | 4.573 | pmm |
| HbA1c | 0.119 | pmm |
| eGFR | 0.278 | pmm |
| Hypertension | 0.199 | polyreg |
| Diabetes | 0.039 | polyreg |

LDL-c, low-density lipoprotein cholesterol; HbA1c, hemoglobin A1c; eGFR, estimated glomerular filtration rate; pmm predictive mean matching, polyreg polytomous logistic regression.
