# Supplementary material for: The association between the metabolic score for insulin resistance and mortality in patients with cardiovascular disease: a national cohort study
Source: Front Endocrinol (Lausanne). 2024 Dec 18;15:1479980. doi: 10.3389/fendo.2024.1479980 (PMC11695433; doi:10.3389/fendo.2024.1479980)
Supplement: Supplementary file 4 [file Table2.docx]

**Table S2.** HRs (95% CIs) for mortality according to the lnMETS-IR quartiles after excluding participants with any missing covariate values at baseline

| Characteristic | Number of deaths | Model 1 |  | Model 2 |  | Model 3 |  |
| --- | --- | --- | --- | --- | --- | --- | --- |
|  |  | HR (95%CI) | P value | HR (95%CI) | P value | HR (95%CI) | P value |
| All-cause mortality | 855 |  |  |  |  |  |  |
| lnMETS-IR (per 1 unit increment) | | 0.54 (0.41, 0.72) | <0.001 | 0.90 (0.67, 1.22) | 0.507 | 0.83 (0.50, 1.39) | 0.481 |
| lnMETS-IR quartile |  |  |  |  |  |  |  |
| Q1 | 245 | 1 |  | 1 |  | 1 |  |
| Q2 | 214 | 0.72 (0.60, 0.87) | 0.001 | 0.72 (0.60, 0.87) | 0.001 | 0.72 (0.59, 0.88) | 0.001 |
| Q3 | 217 | 0.74 (0.62, 0.89) | 0.001 | 0.79 (0.66, 0.96) | 0.015 | 0.79 (0.63, 0.99) | 0.040 |
| Q4 | 179 | 0.68 (0.56, 0.82) | <0.001 | 0.96 (0.79, 1.17) | 0.712 | 1.00 (0.74, 1.33) | 0.975 |
| P for trend |  |  | <0.001 |  | 0.702 |  | 0.755 |
| Cardiovascular mortality | 353 |  |  |  |  |  |  |
| lnMETS-IR (per 1 unit increment) | | 0.57 (0.37, 0.88) | 0.012 | 1.03 (0.64, 1.65) | 0.893 | 1.15 (0.51, 2.59) | 0.739 |
| lnMETS-IR quartile |  |  |  |  |  |  |  |
| Q1 | 99 | 1 |  | 1 |  | 1 |  |
| Q2 | 87 | 0.73 (0.55, 0.97) | 0.031 | 0.72 (0.54, 0.97) | 0.028 | 0.75 (0.55, 1.03) | 0.078 |
| Q3 | 88 | 0.74 (0.56, 0.99) | 0.043 | 0.80 (0.60, 1.07) | 0.137 | 0.88 (0.62, 1.26) | 0.499 |
| Q4 | 79 | 0.74 (0.55, 0.99) | 0.045 | 1.11 (0.82, 1.50) | 0.513 | 1.28 (0.81, 2.00) | 0.290 |
| P for trend |  |  | 0.060 |  | 0.567 |  | 0.381 |

Model 1: no covariates were adjusted;

Model 2: adjusted for age, gender, race;

Model 3: adjusted for covariates in Model 2 plus education level, smoking status, married status, alcohol drinking, BMI, waist circumference, PIR, PA, LDL-c, TC, HbA1c, eGFR, hypertension, diabetes status.

METS-IR, metabolic score for insulin resistance; BMI, body mass index; PIR, family poverty income ratio; PA, physical activity; LDL-c, low-density lipoprotein cholesterol; TC, total cholesterol, HbA1c, hemoglobin A1c; eGFR, estimated glomerular filtration rate; HR, hazard ratio; CI, conﬁdence interval.
