# Supplementary material for: The association between the metabolic score for insulin resistance and mortality in patients with cardiovascular disease: a national cohort study
Source: Front Endocrinol (Lausanne). 2024 Dec 18;15:1479980. doi: 10.3389/fendo.2024.1479980 (PMC11695433; doi:10.3389/fendo.2024.1479980)
Supplement: Supplementary file 5 [file Table3.docx]

**Table S3.** HRs (95% CIs) for mortality according to the lnMETS-IR quartiles after excluding participants with self-reported cancer at baseline.

| Characteristic | Number of deaths | Model 1 |  | Model 2 |  | Model 3 |  |
| --- | --- | --- | --- | --- | --- | --- | --- |
|  |  | HR (95%CI) | P value | HR (95%CI) | P value | HR (95%CI) | P value |
| All-cause mortality | 871 |  |  |  |  |  |  |
| lnMETS-IR (per 1 unit increment) | | 0.60 (0.46, 0.78) | <0.001 | 0.96 (0.72, 1.28) | 0.784 | 0.86 (0.53, 1.38) | 0.524 |
| lnMETS-IR quartile |  |  |  |  |  |  |  |
| Q1 | 240 | 1 |  | 1 |  | 1 |  |
| Q2 | 219 | 0.76 (0.63, 0.92) | 0.004 | 0.77 (0.64, 0.93) | 0.006 | 0.78 (0.64, 0.94) | 0.011 |
| Q3 | 217 | 0.74 (0.62, 0.89) | 0.002 | 0.78 (0.65, 0.94) | 0.009 | 0.78 (0.62, 0.97) | 0.026 |
| Q4 | 195 | 0.73 (0.61, 0.89) | 0.001 | 1.02 (0.84, 1.24) | 0.807 | 1.02 (0.76, 1.36) | 0.900 |
| P for trend |  |  | 0.047 |  | 0.453 |  | 0.885 |
| Cardiovascular mortality | 371 |  |  |  |  |  |  |
| lnMETS-IR (per 1 unit increment) | | 0.66 (0.44, 0.99) | 0.047 | 1.18 (0.76, 1.85) | 0.453 | 0.95 (0.45, 1.99) | 0.885 |
| lnMETS-IR quartile |  |  |  |  |  |  |  |
| Q1 | 97 | 1 |  | 1 |  | 1 |  |
| Q2 | 93 | 0.80 (0.60, 1.06) | 0.125 | 0.82 (0.61, 1.09) | 0.163 | 0.83 (0.61, 1.12) | 0.218 |
| Q3 | 93 | 0.79 (0.59, 1.05) | 0.106 | 0.84 (0.63, 1.12) | 0.238 | 0.85 (0.61, 1.20) | 0.365 |
| Q4 | 88 | 0.82 (0.61, 1.09) | 0.173 | 1.22 (0.91, 1.64) | 0.193 | 1.19 (0.77, 1.84) | 0.446 |
| P for trend |  |  | 0.190 |  | 0.266 |  | 0.657 |

Model 1: no covariates were adjusted;

Model 2: adjusted for age, gender, race;

Model 3: adjusted for covariates in Model 2 plus education level, smoking status, married status, alcohol drinking, BMI, waist circumference, PIR, PA, LDL-c, TC, HbA1c, eGFR, hypertension, diabetes status.

METS-IR, metabolic score for insulin resistance; BMI, body mass index; PIR, family poverty income ratio; PA, physical activity; LDL-c, low-density lipoprotein cholesterol; TC, total cholesterol, HbA1c, hemoglobin A1c; eGFR, estimated glomerular filtration rate; HR, hazard ratio; CI, conﬁdence interval.

**多个回归方程**

| Exposure | Non-adjusted | Adjust I | Adjust II |
| --- | --- | --- | --- |
| ALLDEATH |  |  |  |
| NHHR | 0.90 (0.88, 0.93) <0.0001 | 1.01 (0.98, 1.04) 0.5167 | 0.97 (0.94, 1.00) 0.0598 |
| NHHR 四分组 |  |  |  |
| Q1 | 1.0 | 1.0 | 1.0 |
| Q2 | 0.77 (0.70, 0.86) <0.0001 | 0.86 (0.78, 0.96) 0.0046 | 0.83 (0.75, 0.92) 0.0003 |
| Q3 | 0.74 (0.66, 0.82) <0.0001 | 0.92 (0.83, 1.02) 0.1181 | 0.86 (0.78, 0.96) 0.0066 |
| Q4 | 0.65 (0.59, 0.72) <0.0001 | 0.96 (0.87, 1.07) 0.5115 | 0.84 (0.76, 0.94) 0.0022 |
| NHHR 四分组 | 0.87 (0.84, 0.90) <0.0001 | 0.99 (0.96, 1.03) 0.6683 | 0.95 (0.92, 0.99) 0.0058 |
| CVDDEATH |  |  |  |
| NHHR | 0.92 (0.88, 0.97) 0.0026 | 1.05 (1.00, 1.11) 0.0566 | 1.00 (0.95, 1.06) 0.9875 |
| NHHR 四分组 |  |  |  |
| Q1 | 1.0 | 1.0 | 1.0 |
| Q2 | 0.75 (0.62, 0.91) 0.0039 | 0.86 (0.71, 1.04) 0.1185 | 0.80 (0.66, 0.97) 0.0250 |
| Q3 | 0.77 (0.63, 0.93) 0.0061 | 0.99 (0.81, 1.19) 0.8810 | 0.87 (0.72, 1.06) 0.1742 |
| Q4 | 0.66 (0.54, 0.80) <0.0001 | 1.03 (0.84, 1.26) 0.7734 | 0.85 (0.70, 1.04) 0.1225 |
| NHHR 四分组 | 0.88 (0.83, 0.94) <0.0001 | 1.02 (0.96, 1.09) 0.5540 | 0.96 (0.90, 1.02) 0.2048 |

表中数据：HR (95% CI) Pvalue *P<0.05 **P<0.01 ***P<0.001
结果变量: ALLDEATH; CVDDEATH
暴露变量: NHHR; NHHR 四分组; NHHR 四分组
Non-adjusted model adjust for: None
Adjust I model adjust for: GENDER; AGE; RACE
Adjust II model adjust for: GENDER; AGE; RACE; EDUCATION; MARRY; PIRG; SMOKING; DRINKING; BMI; EGFR; METSG
Cox model 时间变量: TIME
此表用易侕统计软件 (www.empowerstats.com) 和R软件生成，生成日期： 2024-09-20

**多个回归方程**

| Exposure | ALLDEATH | CVDDEATH |
| --- | --- | --- |
| NHHR | 0.99 (0.96, 1.02) 0.6021 | 1.01 (0.96, 1.07) 0.6357 |
| NHHR 四分组 |  |  |
| Q1 | 1.0 | 1.0 |
| Q2 | 0.85 (0.77, 0.95) 0.0029 | 0.81 (0.67, 0.99) 0.0386 |
| Q3 | 0.92 (0.83, 1.03) 0.1633 | 0.91 (0.74, 1.12) 0.3782 |
| Q4 | 0.91 (0.81, 1.02) 0.1164 | 0.89 (0.71, 1.10) 0.2835 |
| NHHR 四分组 | 0.98 (0.94, 1.02) 0.2343 | 0.97 (0.91, 1.04) 0.4468 |

表中数据：HR (95% CI) Pvalue *P<0.05 **P<0.01 ***P<0.001
结果变量: ALLDEATH; CVDDEATH
暴露变量: NHHR; NHHR 四分组; NHHR 四分组
Adjust model adjust for: GENDER; AGE; RACE; EDUCATION; MARRY; PIRG; SMOKING; DRINKING; BMI; CVDS; EGFR; METSG; DM; ANTI.DM; HBP.DRUG; HTC.DRUG; HYPERLIPIDEMIA
Cox model 时间变量: TIME
此表用易侕统计软件 (www.empowerstats.com) 和R软件生成，生成日期： 2024-09-20
